# CaP-Coated Cyclosporine A Liposomes Formulated as an Inhalable Dry Powder for Lung Inflammatory Diseases

**DOI:** 10.3390/pharmaceutics18060684

**Published:** 2026-05-30

**Authors:** Davide D’Angelo, Stefania Glieca, Lisa Flammini, Simona Bertoni, Annalisa Bianchera, Eride Quarta, Ben Forbes, Fabio Sonvico, Francesca Buttini

**Affiliations:** 1 Department of Food and Drug, University of Parma, Parco Area Delle Science 27/A, 43124 Parma, Italystefania.glieca@unipr.it (S.G.); lisa.flammini@unipr.it (L.F.); simona.bertoni@unipr.it (S.B.); annalisa.bianchera@unipr.it (A.B.); eride.quarta@unipr.it (E.Q.);; 2 Institute of Pharmaceutical Science, King’s College London, 150 Stamford Street, London SE1 9NH, UK; ben.forbes@kcl.ac.uk; 3 Interdepartmental Center for Innovation in Health Products, Biopharmanet Tec, University of Parma, Parco Area Delle Scienze 27/A, 43124 Parma, Italy

**Keywords:** cyclosporine A, liposomes for inhalation, calcium phosphate, dry powders for inhalation, lung transplant rejection

## Abstract

**Background:** Cyclosporine is widely used to prevent transplant rejection; however, its systemic administration is associated with low bioavailability and a risk of severe adverse side effects. In the context of lung transplantation, local pulmonary delivery represents a promising strategy to reduce the required dose while enhancing local anti-inflammatory efficacy and limiting systemic toxicity. **Methods:** In this study, cyclosporine was encapsulated in liposomes coated with calcium phosphate to improve cellular uptake. The liposomal formulation was subsequently converted into a dry powder for inhalation to enable pulmonary administration, combining cyclosporine-loaded liposomes with a calcium phosphate coating, extending prior work on inhaled liposomal cyclosporine and mineral-coated liposomes into a single platform. The cyclosporine loading was optimised to achieve an efficient drug content in the final formulation. **Results:** The presence of the calcium phosphate coating on the liposomal surface was confirmed by the shift in zeta potential and by cryo-transmission electron microscopy. The resulting dry powder exhibited suitable aerodynamic properties for pulmonary delivery with a fine particle fraction of 33.6 ± 1.6%. In vitro biocompatibility studies performed on A549 epithelial cells and THP-1 monocytic cells demonstrated that the formulation did not affect cell viability. Furthermore, the formulation containing calcium phosphate-coated liposomes showed a stronger anti-inflammatory effect compared with both uncoated liposomal formulations and the corresponding raw material, consisting of a physical mixture of phospholipids and cyclosporine. **Conclusions:** Overall, despite limitations on respirability and efficacy that will require further in vivo studies, this calcium phosphate-coated liposomal dry powder could represent a promising strategy for targeted pulmonary delivery of cyclosporine, with potential to improve the prevention of lung transplant rejection while minimising systemic side effects.

## 1. Introduction

Since its discovery in 1972, cyclosporine A (CsA) has been widely used in clinical conditions requiring immune system modulation, such as autoimmune and chronic inflammatory diseases and transplant medicine [1]. Its mechanism of action is based on the reversible inhibition of calcineurin, preventing the activation of T cells and therefore the release of pro-inflammatory cytokines, overall modulating the immune response. Its action on T lymphocytes makes it a suitable candidate for the treatment of bronchiolitis obliterans syndrome (BOS), a complication of lung transplantation characterised by inflammation and consequently obstruction of the small airways, responsible for 30% of the deaths in the first year after transplantation [2]. Moreover, CsA has been proposed as a treatment candidate of SARS-CoV-2 infection, in the wake of a number of existing drug repurposing during the COVID-19 pandemic, as its effects on the airways are very broad. CsA can modulate the inflammatory processes in the lungs, preventing the “cytokine storm” responsible for the lung damage in the most severe forms of the disease [3]. Furthermore, CsA interferes with angiotensin II’s harmful effects, including vasoconstriction and thrombotic effects, responsible for atherosclerosis, hypertension and heart failure [3]. Recently, our group has shown that CsA can inhibit virus replication in vitro both as a preventative and post-infection treatment and that this effect was increased when the drug was incorporated into α-tocopheryl-polyethylene-glycol succinate micelles [4].

The challenge in using this 1.2 kDa cyclic polypeptide is that it is very lipophilic and a target of pre-systemic metabolism, which reduces its oral bioavailability to around 30%, leads to high dosing and causes high inter-individual variability [5]. This variability in drug oral bioavailability not only influences the clinical outcome of the treatment, but also poses a severe risk of adverse effects, such as nephrotoxicity, hepatotoxicity, neurotoxicity and hypertension [6]. A formulative approach for increasing the solubility of the drug and limiting the systemic exposure is its direct administration to the target site by using nanostructures, such as micelles or liposomes [5]. Liposomes could represent an appropriate drug delivery system for the lungs since, being composed of phospholipids that comprise pulmonary surfactant [7], they are biocompatible and may be able to evade alveolar macrophage uptake [8]. In addition, these phospholipid-based nanoparticle systems, when administered locally to the lung, avoid drug accumulation in organs such as the liver, kidney, brain and heart, thereby potentially limiting the drug-related toxicity [9]. Because of their unique structure and chemistry they can encapsulate different types of molecules with high efficiency, including lipophilic or biologic drugs [8]. Furthermore, because of their small size (nm), they can easily penetrate the mucus layer and interact with the epithelial surface of the airways, allowing for drug accumulation and retention in the target organ [10]. Different liposomal formulations for inhalation are currently in clinical trials for local pulmonary treatment of infections, with promising data. A highly concentrated amikacin liposomal suspension (Arikayce^®^, Insmed Inc., Bridgewater, NJ, USA) was shown to be effective in refractory nontuberculous mycobacterial lung disease, with reduced systemic toxicity compared to the parenteral administration due to a 274-fold higher drug level in lung tissue [11]. Moreover, nebulised liposomal amphotericin B (Ambisome^®^, Astellas Pharma US, Northbrook, IL, USA; marketed for IV infusion) was reported to be effective both for prophylaxis and topical treatment of fungal infections, without serious adverse effects, probably because no drug was measurable in plasma samples [12].

Phase III clinical studies are currently ongoing to assess the efficacy of a nebulised CsA-liposome formulation to treat chronic lung allograft dysfunction and BOS in patients post single (BOSTON-1) [13] or double (BOSTON-2) [14] lung transplant using a PARI eFlow nebuliser. The clinical studies are supported by previously positive results on the inhalation of this formulation, which suggests that once- or twice-daily dosage of 10 mg CsA is sufficient to obtain a peripheral lung deposition of 14–28 mg/week [15].

However, during storage, liposomes in the liquid formulation can experience the loss of the entrapped drug as well as modifications of their physical properties, such as structural reorganisation and size increase [10]. Furthermore, the shearing occurring in the nebulisers to transform the liposomal dispersion in inhalable aerosol droplets can result in vesicle damage/fragmentation [7]. For this reason, drying of a liposome dispersion using suitable techniques to obtain respirable particles, such as spray drying, can represent an option to overcome the stability issues. In addition, dry powders for inhalation (DPIs) containing liposomes can provide controlled-release properties to the inhalable formulation, which could reduce the frequency of drug administration [8] and increase the compliance of chronically treated patients. The controlled and local release of the drug to the site of action can also be achieved by covering the liposomal surface with biocompatible and biodegradable material such as calcium phosphate (CaP). The negatively charged liposomal surface can be covered by the direct precipitation of a calcium phosphate solution to form a shell that is soluble at acidic pH [16]. This would allow the release of the drug only in the districts with pH < 5, such as lysosomes or inflamed tissues [17]. The improvement of protein-loaded calcium phosphate nanoparticle uptake was demonstrated across various cell lines (HeLa, MG-63, THP-1 and hMSC) [18]. The enhancement of cell uptake due to CaP was also previously reported in lipid nanoparticles coated with CaP that exhibited a significantly higher cellular accumulation in human osteosarcoma U-2OS cells compared to uncoated nanoparticles [19]. In addition, vincristine-loaded liposomes showed a 4-fold higher rate of CaP-coated liposome uptake compared to the uncoated ones in A549 cells [20].

Therefore, this study aims to develop an innovative inhalable CsA powder based on CaP-coated liposomes capable of providing a high drug concentration at the site of action, i.e., the lungs, reducing the possible long-term systemic effects. After selecting the best composition of liposomes in terms of drug encapsulation efficiency and size, the liposomal suspension was spray-dried, adding suitable excipients for improving its respirability. Finally, the tolerability and the anti-inflammatory effect of the liposomal formulation in LPS-treated THP-1/A549 cells co-culture were assessed.

## 2. Materials and Methods

### 2.1. Preparation of CsA-Loaded Liposomes Coated with Calcium Phosphate

Liposomes were prepared by solubilising lecithin from soybean containing 75% phosphatidylcholine (Lipoid S80, Lipoid GmbH, Ludwigshafen, Germany), and, when present, cholesterol (Sigma Aldrich, St. Louis, MO, USA), in ethanol (17.5% *w*/*v*), while CsA (Metapharmaceutical, Barcelona, Spain) was dissolved in a hydroalcoholic solution (33% *v*/*v* ethanol in water). Cholesterol was added with a molar ratio of 30% or 20% to lecithin. The concentrations of CsA in the hydroalcoholic solution were 4.8%, 7.0% and 9.0% *w*/*v* representing Batch A, B and C, respectively (Table 1). Both solutions were heated up to 70 °C, above the glass transition temperature of the lipidic components [19]. Liposomes were obtained using the ethanol injection method, adding drop by drop the lecithin solution to the one containing CsA. Then, liposome dispersions were subjected to twenty cycles of homogenisation at 800 bar using a High-Pressure Homogeniser (HPH) (Panda Plus 2000, GEA Niro Soavi SpA, Parma, Italy) to reduce the particle size and narrow size distribution, without temperature control. After homogenisation, the liposomes were diluted 1:10 with ultrapure water (final ethanol concentration 4.28% *v*/*v*) in a final volume of 97 mL. After this step the liposomes were coated with CaP using a sequential step addition approach, in which a calcium chloride (Sigma Aldrich, St. Louis, MO, USA) solution (50 mM) and a disodium hydrogen phosphate (Sigma Aldrich, St. Louis, MO, USA) solution (50 mM), 1.5 mL each, were added to the liposome dispersion (composition reported in Table 1), reaching a final volume of 100 mL. Finally, the liposomes were dialysed for 24 h using a Spectra/Por^®^ 3 dialysis membrane with a 3.5 kDa MW cut-off (SpectrumLabs, Repligen, Waltham, MA, USA) in a hydroalcoholic (4.28% *v*/*v* EtOH) medium to eliminate unreacted calcium chloride and disodium hydrogen phosphate.

### 2.2. Drug Content Quantification and Solid Quantification in Liposomal Formulations

CsA quantification was performed using a validated HPLC-UV method and using a C18 column (4 µm, 3.9 × 150 mm) (Nova-Pak, Waters Technologies Corporation, Wexford, Ireland) maintained at 65 °C. For CsA detection a UV detector set at 230 nm was used. The mobile phase consisted of a solution of acetonitrile and water in a 63:35 *v*/*v* ratio [4,21]. All the samples were solubilised in acetonitrile and water solution (80:20 *v*/*v*) and sonicated for 5 min to ensure the disruption of the liposomal structure. The solid content in the liposomal suspension was obtained by drying 2 mL of dispersion for 24 h in an oven with a controlled temperature of 40 °C. The solid content was expressed as mg/mL. The encapsulation efficiency (EE%) was calculated as the ratio between the total amount of CsA recovered in liposomes and the initial amount of drug employed for the preparation, expressed in percentage.

### 2.3. Calcium Titration in Coated Liposomes

The presence of calcium phosphate in the formulation, after the dialysis process, was determined by calcium back-titration as described in the USP-NF 2023 [22]. CaP-coated liposomes (40 mL) were treated with 1.2 mL of HCl 0.1 M solution to dissolve the CaP coating. An excess of EDTA was added to the sample solution. The solution was buffered using NH_4_Cl/NH_3_ to a pH of 10. Eriochrome Black T (Carlo Erba, Milano, Italy) was used as an indicator. A solution of ZnSO_4_ (0.02 M) was used as a titrant to determine moles of unbound EDTA. The volume of EDTA needed to bind the Ca^2+^ in the sample was obtained from the difference between the volume of ZnSO_4_ and the total amount of EDTA added to the solution. Because EDTA binds calcium with a stoichiometric ratio of 1:1, the moles of calcium in the solution were calculated [22].

### 2.4. Size and Zeta Potential of the Liposomes

The size of the liposomes and their distribution in the dispersion, considering the value of the polydispersity index (PDI), as well as the ζ-potential of the liposomal dispersion, were analysed via Non-Invasive Back-Scatter Dynamic Light Scattering (NIBS-DLS) and Electrophoretic Light Scattering (ELS) using a Zetasizer Nano (Malvern Panalytical, Malvern, UK) at 25 °C. For the measurement of uncoated liposomes, a refractive index typical for phospholipids of 1.45 was selected, while for coated liposomes a value of 1.63 was used considering the value of hydroxyapatite (HA) as a reference material. The size measurements were performed with a back-scatter detection of 630 nm and 173° back-scatter angle in both cases. Since water was chosen as the dispersion medium, a refractive index of 1.33 and viscosity of 0.887 cP were determined. Then, the dispersion was diluted with ultrapure water at a concentration of about 20 µg/mL to reach an optimal signal quality measurement. The diluted dispersion was slowly introduced into a cuvette (ZEN0040 in polystyrene, 10 × 10 × 45 mm, Sarstedt AG & Co., Nümbrecht, Germany) to avoid the formation of air bubbles. The run analysis duration was 10 s, while the equilibration time was 120 s.

The liposome redispersion study was assessed by NIBS-DLS analysis by dissolving 10 mg of the spray-dried powder either in 10 mL of phosphate-buffered saline (PBS, pH = 7.4) simulating lung fluids or sodium acetate buffer (pH = 5) simulating inflammatory or endosomal conditions.

For ζ-potential measurements, liposome samples were diluted in a low-ionic-strength electrolyte solution. A 1 mM KCl solution was prepared using Milli-Q water and pre-filtered (0.2 µm) prior to use. Liposomes were diluted at a 1:33 (*v*/*v*) ratio by mixing 30 µL of liposomal suspension with 970 µL of 1 mM KCl, yielding a final lipid concentration of 0.3 mg/mL.

To remove potential dust particles or large aggregates, the diluted suspension was passed through a 0.45 µm syringe filter. The sample was then allowed to rest for 2–5 min at room temperature to eliminate air bubbles before analysis. Approximately 200 µL of the prepared sample was loaded into a disposable folded capillary zeta cell DTS1070 (Malvern Panalytical, Malvern, UK) for electrophoretic light scattering measurements. Samples were equilibrated at 25 °C for 5–10 min prior to acquisition. Zeta potential measurements were performed in triplicate, with each replicate consisting of 15 runs measured using the Smoluchowki method for aqueous solution. For each measurement the conductivity of the diluted sample was recorded.

Three measurements at 25 °C for each sample were performed.

Liposomes size and ζ-potential were evaluated after sample preparation, homogenisation, and CaP coating and dialysis.

### 2.5. Cryo-Transmission Electron Microscopy

Cryo-transmission electron microscopy (cryo-TEM) analysis was performed to visualise liposomes in their native hydrated state, allowing the observation of the coating while preserving their structural integrity and avoiding artefacts associated with drying procedures typical of other electron microscopy techniques such as SEM and traditional TEM. Images of unfixed frozen hydrated liposomal samples were acquired at the laboratories of the Department of Life Sciences of the University of Siena (Italy). Before the analysis, 2.3 µL of sample was placed onto a Quantifoil^®^ copper grid and frozen using a Vitrobot Mark IV (FEI, ThermoFisher Scientific, Wilmignton, NC, USA) instrument with blot force -2 and blotting time 3 s at 20 °C and 100% humidity. The samples were imaged using a CM200 FEG (transmission electron microscope FEI, ThermoFisher Scientific) operating at 200 kV, equipped with a TemCam F224HD (TVIPS GmbH, Gilching, Germany) and cryo-transfer holder 626 DH (Gatan Inc., Pleasanton, CA, USA).

### 2.6. Spray-Dried Microparticles Embedding CsA Liposomes and Morphological Analysis

Spray-dried microparticles for inhalation embedding CsA-coated liposomes were manufactured using a Mini Spray Dryer B-290 (Büchi, Flawil, Switzerland). The spray drying operating parameters were fixed as follows: inlet temperature 140 °C, air flow rate 742 L/h, aspiration 35 m^3^/h, nozzle 0.7 mm and feed rate 3.5 mL/min. Seven spray-dried powders were obtained: four powders adding mannitol in a ratio from 1:1 to 1:4 with respect to the solids in the coated liposome dispersion (LipoM-CsA_1, LipoM-CsA_2, LipoM-CsA_3, LipoM-CsA_4, respectively); and the remaining three powders prepared keeping fixed the 1:3 mannitol ratio and adding either L-leucine (15% *w*/*w*) to the coated (LipoM_Leu-CsA) or uncoated (ULipoM_Leu-CsA) liposome dispersion or sodium stearate (0.5% *w*/*w*) (LipoM_NaSt-CsA). Depending on the composition, in all the cases the solid concentration was less than or equal to 1.1% *w*/*w*. The yield of the process was calculated as the percentage ratio between the powder recovered from the spray drying and the total solids in the dispersion before the drying.

Particle morphology was determined by SEM (Zeiss AURIGA, Zeiss, Oberkochen, Germany) performed under high-vacuum conditions with an accelerating 1.0 kV voltage, at 5 k times magnification. Powders were deposited on adhesive black carbon tabs pre-mounted on aluminium stubs and imaged without undergoing any metallisation process.

### 2.7. Aerodynamic Characterisation

For the respirability analyses, a Next Generation Impactor (NGI, Copley Scientific Limited, Nottingham, UK) was employed. The air flow was set at 65 L/min and the duration at 3.7 s to obtain an air passing volume of 4 L. For the analysis, an RS01^®^ (Plastiape, Italy) high-resistance device was employed to aerosolise about 20 mg of spray-dried powders used to fill HPMC extra-dry Quali-V^®^-I size #3 capsules (Qualicaps, Madrid, Spain). The powder remained in the device, and that deposited in the induction port (IP), stages and Micro Orifice Collector (MOC), was collected using an ACN:H_2_O solution (65:35) acidified with HCl (1% *w*/*v*) to pH 4. The samples were analysed using the validated HPLC method, and the respirability parameters were calculated. In detail, the dose of CsA leaving the device after the aerosolisation represented the emitted dose (ED). A distribution curve was obtained by plotting the cumulative percentage of mass less than the stated size cut-off for each NGI stage on a probability scale versus the cut-off diameter of the respective stage on a logarithmic scale. A log-probability plot equation allowed the calculation of the aerodynamic parameters: the mass median aerodynamic diameter (MMAD), and the fine particle mass (FPM), representing the dose of powder with an aerodynamic diameter < 5 µm and the extra-fine particle mass (EFPM) < 2 µm. The fine particle fraction (FPF) and the extra-fine particle fraction (EFPF) were expressed as the percentage ratio between the FPM and EPFM and the ED, respectively.

### 2.8. Viability Study on A549 and THP-1 Treated with Liposomal Formulations

Cytotoxicity of the CsA-loaded liposomes was investigated by determining cell viability of two relevant human cell lines, i.e., A549 human alveolar epithelial cells and THP-1 human monocyte–macrophage cells. For the cytotoxicity studies, A549 cells were seeded at a concentration of 1.25 × 10^5^ cells/mL. THP-1 cells were seeded at a concentration of 6 × 10^5^ cells/mL in 96-well plates at 37 °C.

Cells were exposed for 24 h [23] to the following treatments: vehicle (RPMI-1650 + 2% FBS), mannitol 2.5 μg/mL, CsA raw material 1 µg/mL in 0.5% DMSO (*v*/*v*) (CsA_rm), a physical mixture of phospholipids 10 µg/mL and CsA raw material 1 µg/mL (PL + CsA), spray-dried powder containing mannitol, leucine and CsA CaP-coated liposomes (LipoM-Leu-CsA), and powder containing mannitol, leucine and CsA CaP-uncoated liposomes (ULipoM-Leu_CsA). Both powders were suspended to obtain a CsA concentration of 1 µg/mL. The final concentration of CsA was selected according to a previous study [21], while the concentrations of mannitol and phospholipids were determined according to their relative ratios to CsA in the formulation.

Cell viability was quantified using the MTS assays [24]. Briefly, 20 μL MTS (3-(tributylammonium)-propyl methanethiosulfonate bromide solution) (1 mg/mL) was added to each well and, following 4 h incubation at 37 °C, the supernatants were collected. The absorbance/well was measured at 490 nm (Sunrise™ powered by Magellan™ data analysis software, TECAN, Mannedorf, Switzerland).

The impact of the various treatments on cell viability was expressed as the percentage of cell viability with respect to the value obtained using only the vehicle.

### 2.9. Co-Culture Assays and IL-6 Determination

On Transwell^®^ culture plates (Corning 3470), A549 cells (10^5^ cells/well) were seeded at the bottom, and THP-1 cells (10^5^ cells/well) were plated on the insert (0.4 μm pore polyester filter). The two cell cultures were physically separated to avoid their direct contact according to the method described by Li et al., 2020 [25]. After 24 h co-culture, the following treatments were applied to the lower compartment: vehicle, mannitol 2.5 μg/mL, CsA_rm, PL + CsA, LipoM-Leu-CsA, and ULipoM-Leu-CsA.

One hour after the treatment, cells were exposed for 24 h to lipopolysaccharide from E. coli O55:B5 (Sigma-Aldrich, St. Louis, MO, USA) at a concentration of 1 µg/mL. Cells incubated with vehicle and not exposed to LPS were used as a negative control. The concentration of IL-6 in the conditioned media was subsequently analysed using a human IL-6 (interleukin 6) ELISA kit (Wuhan Fine Biotech Co., Ltd., FineTest^®^, Wuhan, Hubei, China), according to the manufacturer’s protocol and expressed as percentage relative to LPS-treated control.

Statistical analysis was carried out using one-way analysis of variance (ANOVA) to evaluate differences among groups followed by the Holm–Šidák multiple comparison test. Differences were considered statistically significant at a *p*-value ≤ 0.05.

All experiments were performed in triplicate (*n* = 3), and data are expressed as mean ± standard deviation.

## 3. Results and Discussion

### 3.1. Liposomal Formulation Development and Characterisation

The formulation of poorly soluble drugs in liposomes has several advantages, such as biocompatibility, the possibility to tailor the composition of the liposomal membrane to modify the drug release rate, and the possibility to produce them in different size ranges [26]. For pulmonary administration, size is of primary importance as it influences the interaction with airway epithelia, absorption and consequently the bioavailability of the drug [27]. Although the most effective particle size for absorption at the alveolar level has not yet been defined, nanoparticles measuring less than 150 nm are reported to have reduced lung clearance and higher transepithelial transport compared to larger particles [27]. In addition, because nanoparticles are deposited in higher concentrations by number compared to microparticles, their phagocytic clearance is delayed [28]. However, a safe, stable and efficient liposome formulation is characterised by a homogenous and monodisperse population of nanoparticles, expressed by the PDI value. Although FDA industry guidance does not indicate a reference range [29], it is customary to indicate a PDI lower than 0.3 for liposomal systems as indicative of a homogenous monodisperse system [27].

For the preparation of liposomes, different CsA concentrations and the addition of cholesterol were investigated to identify the best conditions to obtain stable liposomes, with a size suitable for the target application that would allow to encapsulate a high amount of drug. In Table 2 the sizes of liposomes obtained after the different steps of the production are reported.

Immediately after production, the liposomes’ size ranged between 300 and 400 nm with a very high PDI, outside the acceptability range, despite their composition. The high-pressure homogenisation process allowed a drastic reduction in the size of liposomes to 40–60 nm and a more homogeneous size distribution, which resulted in a PDI lower than 0.3 for all the batches. After CaP coating and dialysis purification, a reduction in the diameter of the liposomes in Batches B and C was further observed. This is reasonable considering that, during the purification process, the material that did not take part in the formation of the liposomes and that could interact with the liposomal structures through weak interactions was eliminated. The medium exchange occurring during dialysis, in fact, can increase the electrostatic repulsion, reducing the possibility of particle fusion, and lead to a reduction in the particle size distribution of the vesicles [30].

The addition of cholesterol to the composition of the liposomes (Batches D and E) was investigated as it can affect the fluidity of the membrane, improving the overall stability of the vesicles, and it can sustain the release of the drug [31]. Since a high concentration of cholesterol interferes with the close packing of phospholipid bilayers, molar ratios of 20 and 30% with respect to the amount of phospholipids were studied, which corresponded to the maximum amount at which a high encapsulation efficiency was reported [31]. However, after coating and dialysis, an increase in the size of liposomes from 40 nm to 100 nm and a very high PDI (>0.4) were observed. Furthermore, this increase in liposome size was correlated with an increase in the molar concentration of cholesterol used for the preparation of the liposomes, a behaviour already observed in a previous study [32]. However, in our case, the preparation of liposomes with cholesterol led to the formation of large aggregates that showed a tendency to crystallise and precipitate, possibly related to a cholesterol-related reduction in CsA capability to intercalate phospholipid bilayers as reported in the literature [33].

For all the batches, the final **ζ**-potential values for the liposomes after coating were less negative compared to the initial ones, confirming an actual surface modification of the nanostructures after the CaP coating. The **ζ**-potential values were reduced in every batch of 20–30 mV, i.e., from −68/−43 mV before the coating to about −29/−15 mV after coating and dialysis. A reduction in **ζ**-potential value was also reported by Thakkar and colleagues after using a CaCl_2_ and K_2_HPO_4_ solution for the coating of vincristine liposomes [20]. Moreover, a colloidal system with **ζ**-potential in the range of −30/−40 mV is considered stable [34]. In this phase of the study, however, the parameter was mainly interpreted as an effect of the successful coating rather than a quality criterion.

During the formation of liposomes, lipophilic drugs tend to be encapsulated in the phospholipidic tail domain of the bilayer, from which they can be released at different rates depending on the composition of liposomes [26]. However, factors related to the drug characteristics, such as the capacity to interact with the membrane bilayer, or to the liposome properties, such as the membrane rigidity, the surface area and the preparation method, can influence the encapsulation efficiency [35]. The encapsulation efficiency (EE) has been investigated after coating and dialysis of the liposomes for all the batches, and the results are reported in Table 3.

After dialysis, the concentration of total solids determined after evaporation of the solvent was about 2–2.3 mg/mL. Concerning the batches without cholesterol (Batches A–C), the maximum percentage of CsA that was encapsulated in the liposomes was 0.20 mg/mL, with a maximum of 8.6% *w*/*w* (Batch B). When a larger amount of CsA was employed for the preparation of Batch C, which exceeded its solubility in the medium (corresponding to an initial concentration of 9% *w*/*w*), a drug crystal precipitation was observed immediately after the lecithin solution addition. The components that did not take part in the formation of the structures were removed during the dialysis, leading to a drastic reduction in the solid concentration (1.57 mg/mL) and of the EE% (27%).

The addition of cholesterol to the liposome composition could reduce the EE when encapsulating a lipophilic drug because they compete for the same space, so the drug could be displaced by cholesterol as mentioned above [36]. However, this did not happen in our case, as an EE% of about 90% was reached for both batches with cholesterol (Batches D and E). Probably, the amount of cholesterol employed was relatively low and this did not alter the encapsulation capacity of the liposomes, as reported in another study [31].

Notwithstanding, according to the drug content and the overall quality results, Batch B was selected as the lead formulation to be included in an inhalation powder and to be studied in the following experiments.

The successful quantification of calcium after dialysis ensured that, even after this purification process, calcium phosphate was a structural part of the formulation, presumably as a vesicle coating. For this reason, a morphological analysis by cryo-TEM was conducted to visualise in detail the structure of the liposomes produced and confirm the deposition of CaP on the liposome surface. Cryo-electron microscopy techniques allow to obtain images of the nanostructures in their “native” aqueous environment, limiting the artefacts usually occurring when samples are dried before EM imaging [37].

**Figure 1:**
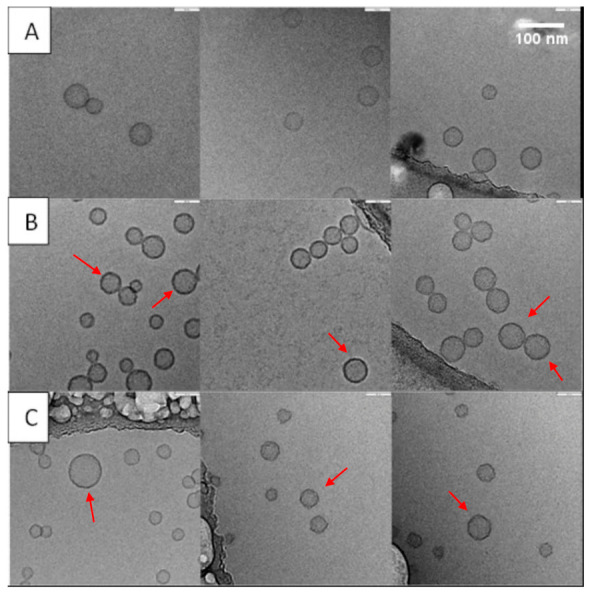
Cryo-TEM images of uncoated liposome (**A**), CaP-coated liposomes (**B**) and cholesterol-containing CaP-coated liposomes (**C**). Arrows indicate the CaP coating on liposomes’ surface.

Figure 1 illustrates cryo-TEM images of CsA-loaded liposomes, liposomes (Batch B) without a coating (A) and CaP-coated (B), and CaP-coated liposomes also containing cholesterol with a molar ratio of 20% with respect to lecithin (Batches E and C).

All the formulations analysed were characterised by liposomes with a unilamellar spherical structure. The uncoated liposomes (Figure 1A) were less visible but had a smooth, regular surface compared to the CaP-coated liposomes (Figure 1B). In the latter formulation, two aspects indicate that they are covered by calcium phosphate salt: the margins are markedly visible and small aggregates of two or more liposomes are more evident. Probably, because the coating changed the surface charge, resulting in a less negative **ζ**-potential, the attractive forces are stronger, resulting in the formation of groups or pairs of liposomes [34]. Moreover, the cryo-TEM images confirmed the PDI results from DLS analysis for the cholesterol-containing liposomes (Figure 1C): the liposomal suspension was characterised by a greater heterogeneity in size compared to the other two samples.

### 3.2. Embedding Coated Liposomes in Microparticles for Inhalation

Despite the numerous advantages of formulating drugs for local delivery in liposomes, liquid formulations are not stable upon storage due to fusion, aggregation or physicochemical modification of lipids [38]. In addition, when liposomes are delivered to the lungs by nebulisation, a shear stress is applied to the formulation, which can cause drug leakage [38]. The drying of liposomes in the form of a powder using spray drying can represent an option to obtain a stable formulation with high aerosolisation efficiency, which also allows the restoration of the liposomes after rehydration. However, during the processing, the vesicles experience mechanical and osmotic stress, which can result in rupture or rearrangement in larger-sized vesicles. Thus, the use of protective excipients is necessary, which replace the water molecules in the drying process of the vesicles, preventing liposomes from collapsing [39]. Mannitol was selected in this study, being an excipient approved for inhalation capable of forming hydrogen bonds and of protecting biologics during the spray drying process, as previously demonstrated by our group [21].

Since Batch B was the most promising, considering the size and the PDI of the liposomes and being the one that allows the maximum loading of the drug into the nanostructures (Table 3), it was chosen to obtain the spray drying powders. In order to optimise powder characteristics both in terms of liposome stability and aerosolisation properties, different mass ratios between liposomes and mannitol were tested (Table 4). Ideally, the ratio should be kept as low as possible to allow for a high concentration of the drug in the formulation. However, the lowest ratios (1:1 and 1:2, corresponding to the powders LipoM-CsA_1 and LipoM-CsA_2) did not allow the construction of suitable microparticles as they tended to deposit in the drying chamber and the cyclone of the spray dryer, leading to production yields lower than 10%. This behaviour was previously reported with spray-dried CaP nanoparticles, where at least a ratio of 1:4 (nanoparticles:mannitol) was required to produce respirable microparticles and to preserve the size of the released nanoparticles [40]. Also in our case, when the amount of mannitol was increased in LipoM-CsA_3 and LipoM-CsA_4, a reduction in adhesive characteristics of the spray-dried powder was obtained with a process yield higher than 60%. In order to limit the amount of excipient to be inhaled, the 1:3 ratio appeared to be a good compromise between the favourable properties of the powder and a high CsA loading (1.59% *w*/*w*).

To improve the respirability and the flowability of this powder, two common excipients used as lubricants, i.e., leucine and sodium stearate, were considered and included in the powders tested. Keeping the liposomes:excipients ratio at 1:3, leucine at 15% *w*/*w* (LipoM_Leu-CsA) and sodium stearate at 0.5% *w*/*w* (LipoM_NaSt-CsA) of the total mass of mannitol were added. Also for these powders the yield of the spray drying was higher than 60%, and the drug content was around 1.7% *w*/*w*.

With the same composition of LipoM_Leu-CsA, a spray-dried powder containing uncoated liposomes (ULipoM_Leu-CsA) was prepared as a control for subsequent investigations.

**Figure 2:**
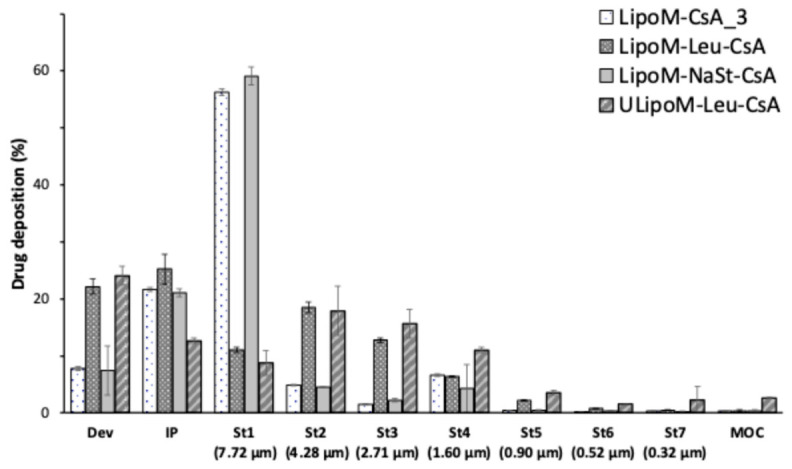
Distribution of embedded coated liposomes in mannitol (LipoM-CsA_3), mannitol and leucine (LipoM-Leu-CsA), mannitol, leucine and sodium stearate (LipoM-NaSt-CsA), and mannitol and leucine embedding uncoated liposomes (ULipoM-Leu-CsA) in a Next Generation Impactor. The loaded amount of powder in the capsule was 20 mg containing 0.32 mg of CsA (*n* = 3, mean ± st.dev.) Dev = device; IP = induction port; St = stage; MOC = Micro Orifice Collector.

The aerosolisation performance of the powders just mentioned was tested in a Next Generation Impactor using a commercially available RS01 device after loading 20 mg of the powders in HPMC size 3 capsules. Results are summarised in Figure 2 and Table 5.

When only mannitol was used as an excipient to obtain the powder (LipoM-CsA_3), although the emitted fraction (EF) was high (92%, Table 5), most of the powder tended to deposit in the first stage of the impactor, leading to an MMAD of 9 µm. The addition of sodium stearate did not improve the aerodynamic behaviour of the powder, which showed a performance almost unchanged compared to the powder without lubricants, with a very low fraction of fine particles (about 2.5%).

On the other hand, the addition of L-leucine to the powder allowed it to improve its aerodynamic behaviour. L-leucine is usually added to spray drying formulations to increase the dispersibility and aerosolisation of the particles [41], and here this ability can be seen in the higher deposition on stages 2 and 3 of the impactor (Figure 2). This led to an increase in the FPF to 33% and a reduction in MMAD to 4 µm (Table 5).

**Figure 3:**
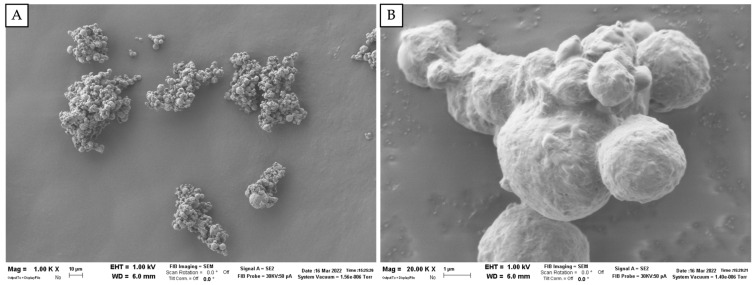
SEM images of the spray-dried powder LipoM_Leu-CsA, containing liposomes embedded in mannitol and leucine at different magnifications; (**A**): scale bar 10 µm, 1 × 10^3^; (**B**): scale bar 1 µm, 2 × 10^4^.

Interestingly, the spray-dried powder containing uncoated liposomes (ULipoM_Leu-CsA) performed better than the one containing CaP-coated liposomes. The former was, in fact, characterised by an FPF of about 50% and an MMAD of 4 µm. Probably, the prevalence of attractive forces between the coated liposomes, demonstrated by Z-potential values closer to zero and by cryo-TEM images, could be responsible for the formation of less redispersible aggregates. The morphological analysis of the LipoM_Leu-CsA powder conducted by SEM confirms that the microparticles are fused to each other, creating aggregates of 4–7 µm (Figure 3). However, given the nonetheless positive respirability results (FPF of 33.6%) and, most importantly, the multiple advantages associated with the calcium phosphate-based coating as discussed above, this powder was selected as the lead formulation for further characterisation. 

### 3.3. Liposome Redispersion

The spray drying process, as already mentioned above, could be responsible for liposome aggregation phenomena, resulting in a larger size when the powder dissolves in vivo. For this reason, although the powder containing leucine has shown acceptable in vitro respirability values, it is appropriate to investigate if the formulation can release the liposomes at their original size. Table 6 illustrates the particle size values of LipoM_Leu-CsA liposomes released after the dissolution of the powder in two aqueous media at different pH levels.

Following redispersion, irrespective of the buffers employed, an increase in the original size of liposomes and PDI could be detected (Table 6). The Z-potential also experienced substantial variations from the starting value. However, under these conditions, it was possible to explain some differences observed between the two buffers on the presence of the CaP coating of the liposomes. A greater value is observed in the size of the liposomes resuspended in acidic conditions, while a smaller value is observed for the liposomes resuspended in a physiological pH buffer. This could be due to a greater solubilisation of the coating at pH 5 with consequent release of Ca^2+^ ions, and a greater aggregation of the nanoparticles as demonstrated also from the reduction in the surface charge from Z-potential values.

### 3.4. In Vitro Toxicity and Anti-Inflammatory Studies

An in vitro toxicity study was carried out to investigate whether the formulation could affect the viability of lung cell lines. The formulation is composed of mannitol, approved by the FDA for inhalation [42], and phosphatidylcholine, the main component of lecithin, a surfactant produced also by type II alveolar cell lines [43]. The studies were carried out on A549 alveolar basal epithelial cells and macrophages (THP-1), employed in the subsequent investigation of the inflammatory process.

**Figure 4:**
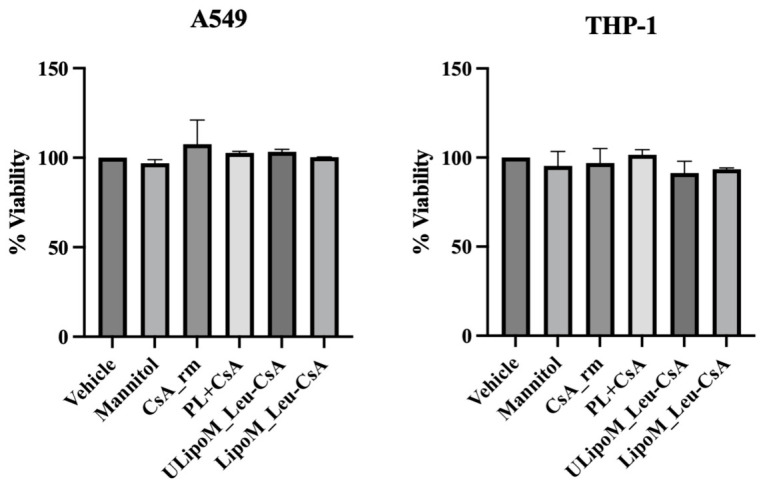
Viability of A549 and THP-1 cell cultures exposed to vehicle, mannitol 2.5 μg/mL, CsA raw material (CsA_rm), a mixture of phospholipids and CsA_rm (PL + CsA), ULipoM_Leu-CsA (uncoated liposomes containing CsA 1 μg/mL), and LipoM_Leu-CsA (coated liposomes containing CsA 1 μg/mL). Data are expressed as percentage with respect to vehicle (mean ± standard deviation, *n* = 3 independent experiments).

The MTS test on A549 and THP-1 cell monocultures showed that the viability of cells was not modified by any of the treatments applied: in particular, when cells were treated with the dissolved LipoM_Leu-CsA spray-dried powders containing CsA, either coated or uncoated, no changes in the viability could be detected (Figure 4).

**Figure 5:**
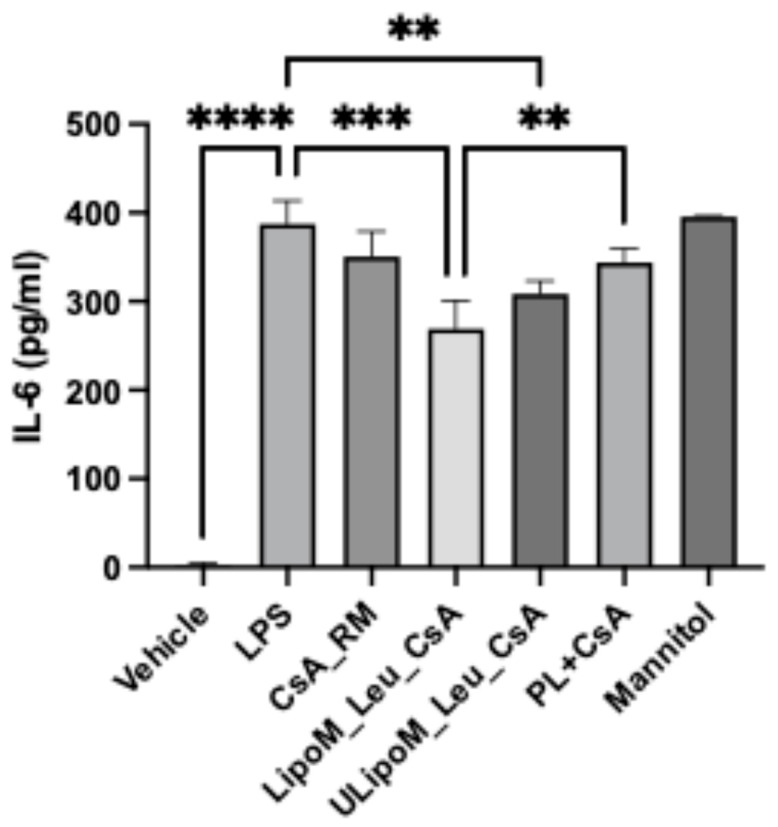
IL-6 production by A549/THP-1 co-culture, exposed to LPS 1 μg/mL, in the presence of vehicle, mannitol, CsA raw material (CsA rm), LipoM_Leu_CsA (coated liposomes) and ULipoM_Leu_CsA(uncoated liposomes), mixture of phospholipids and CsA_rm (PL + CsA). All the CsA-containing formulations and CsA raw were tested at a final concentration of 1 µg/mL. Values are expressed as a percentage with respect to IL-6 levels released by LPS (mean ± standard deviation, *n* = 3). ** *p* < 0.01, *** *p* < 0.001, **** *p* < 0.0001.

The treatment with LPS 1 µg/mL evoked a strong inflammatory response in the A549/THP-1 co-culture, quantified as IL-6 released in the medium (Figure 5). The treatment with only mannitol did not reduce the inflammation, which was comparable to that induced by LPS.

Among CsA-based treatments, exposure to CsA raw material did not significantly reduce IL-6 levels compared to LPS, likely due to its poor solubility in aqueous media. The efficacy of CsA was not improved by the physical mixture with phospholipids, as well, since, despite a potential increase in solubility, it did not provide an improvement in cell penetration and molecular action of the drug. On the other hand, both coated and uncoated CsA-loaded liposomes were able to lower the release of IL-6 induced by LPS, supporting the hypothesis that these lipidic structures can increase the uptake of poorly soluble lipophilic drugs by means of membrane fusion mechanisms. Moreover, CaP-coated liposomes were able to remarkably decrease the production of the inflammatory cytokine also with respect to the physical mixture, highlighting the higher efficacy of this formulation compared to the uncoated one (Figure 5).

Consistent with previous findings reported by Thakkar et al., the CaP coating led to an increase in cellular uptake, potentially leading to a higher intracellular concentration. The study of vincristine-loaded liposomes in A549 showed a 4-fold higher rate of CaP-coated liposome uptake compared to the uncoated [20].

Enhanced cellular uptake due to CaP was also previously reported in the literature on positively or negatively charged CaP-coated lipid nanoparticles, which showed a significantly higher concentration-dependent cellular accumulation of the model red dye in human osteosarcoma U-2OS cells compared to uncoated nanoparticles [19].

The enhancement in cellular uptake observed for CaP-coated liposomes can be explained by several complementary mechanisms. First, the deposition of calcium phosphate onto the liposomal surface alters the physicochemical properties of the particles, particularly their surface charge and interfacial composition. Conventional liposomes often carry a net negative charge, which may limit their interaction with negatively charged cell membranes due to electrostatic repulsion. The presence of CaP can partially shield or modulate this surface charge, reducing repulsion and facilitating closer membrane–particle contact, thereby promoting adsorptive endocytosis. Second, calcium phosphate is intrinsically pH-responsive. While relatively stable at physiological pH, it dissolves under mildly acidic conditions such as those encountered in the endosomal compartment (pH 5–6) [44]. The proton-mediated dissolution of CaP leads to the release of Ca^2+^ and phosphate ions, increasing local osmotic pressure within endosomes and contributing to membrane destabilisation [45]. This mechanism enhances endosomal escape and cytoplasmic release of the encapsulated drug, which may experimentally translate into a higher apparent intracellular accumulation [46]. Additionally, the released Ca^2+^ ions may transiently elevate intracellular calcium levels, influencing membrane trafficking and endocytic pathways, as calcium ions are well-known regulators of vesicular transport and membrane fusion processes [47]. Therefore, the CaP coating not only improves the initial interaction of liposomes with the cell membrane but may also enhance intracellular processing after internalisation.

Taken together, these findings indicate that calcium phosphate coating enhances cellular uptake of liposomes through a combination of surface charge modulation, improved membrane interaction, and pH-triggered intracellular activation. This multifactorial mechanism likely explains the increased intracellular accumulation observed for CaP-coated liposomal formulations. A further investigation was conducted in order to ascertain whether the effective reduction in inflammation by liposomal CsA compared to the physical mixture of CsA and phospholipids could be attributed to its organised lipid structure [37]. Indeed, when phospholipids mixed with CsA were applied to the cells, they did not lead to any reduction in inflammation with respect to LPS (Figure 5).

Our study demonstrated, therefore, that the reduction in inflammation was statistically significant only when phospholipids were organised in CaP-coated liposomal structures loaded with CsA, suggesting that the observed effect resulted from the combined action of the drug and the liposomal carrier, which presumably improved cellular internalisation.

## 4. Conclusions

The current study deals with the production and characterisation of an inhalation powder consisting of CsA loaded in calcium phosphate-coated liposomes with the aim of achieving a formulation able to increase the release of the immunosuppressive drug at the target site and its internalisation at the cellular level. Given that one of the possible therapeutic applications of CsA is to prevent lung transplant rejection, in order to minimise its systemic exposure and promote its internalisation, one option is to entrap the drug in a nanocarrier. The optimised liposomal formulation with the highest percentage of drug was selected, and an inhalable powder with increased stability with respect to the liquid formulation was obtained by spray drying. The in vitro data on the co-culture of alveolar epithelial cells and macrophages showed the high tolerability and anti-inflammatory action of both the coated and uncoated CsA-loaded formulations, highlighting the advantageous effects of CaP-coated liposomes with respect to the non-coated ones and supporting the hypothesis that covering these lipidic structures with CaP can increase the uptake of poorly soluble lipophilic drugs. This study, therefore, represents a first step towards the development of a formulation capable of overcoming key limitations of liposome-based liquid systems. Further studies will be required to confirm these findings in more complex models as well as to assess the translational potential of this approach and its long-term stability.

## Figures and Tables

**Table 1:** Summary of the quali-quantitative composition of CsA liposomes coated with CaP before the dialysis.

	Liposomes Composition				
Batch (CsA Initial Concentration)	CsA (mg/mL)	Lecithin (mg/mL)	Cholesterol (mg/mL)	Na_2_HPO_4_ (mg/mL)	CaCl_2_ (mg/mL)
**A** **(4.8% *w*/*w*)**	0.14	2.54	-	0.13	0.11
**B** **(7.0% *w*/*w*)**	0.21	2.54	-	0.13	0.11
**C** **(9.0% *w*/*w*)**	0.29	2.54	-	0.13	0.11
**D** **(7.0% *w*/*w*)**	0.21	1.77	0.40	0.13	0.11
**E** **(7.0% *w*/*w*)**	0.21	2.01	0.27	0.13	0.11

**Table 2:** Median hydrodynamic particle diameter, polydispersity index (PDI) and surface charge of CsA liposomes after production, after the use of a High-Pressure Homogeniser (HPH) and after the coating process with CaP and dialysis obtained by DLS.

	Initial Dispersion		After Homogenisation			After Coating and Dialysis		
Batch	Size (nm)	PDI	Size (nm)	PDI	ζ-Potential (mV)	Size (d.nm)	PDI	ζ-Potential (mV)
A	291.3 ± 35.1	0.422 ± 0.01	40.0 ± 13.2	0.275 ± 0.09	−51.9 ± 11.8	44.7 ± 12.2	0.168 ± 0.04	−18.6 ± 6.2
B	330.5 ± 27.4	0.472 ± 0.10	67.4 ± 11.9	0.275 ± 0.08	−58.1 ± 8.6	43.3 ± 5.8	0.290 ± 0.22	−27.9 ± 10.3
C	299.3 ± 17.3	0.306 ± 0.02	67.1 ± 39.4	0.191 ± 0.07	−55.5 ± 0.1	47.9 ± 4.3	0.431 ± 0.30	−29.4 ± 0.5
D	454.2 ± 34.3	0.256 ± 0.01	45.9 ± 3.6	0.188 ± 0.04	−43.5 ± 7.1	114.5 ± 4.8	0.431 ± 0.06	−15.1 ± 0.7
E	405.1 ± 55.6	0.420 ± 0.06	46.6 ± 6.1	0.190 ± 0.02	−51.3 ± 3.1	91.7 ± 13.5	0.430 ± 0.04	−18.4 ± 0.7

**Table 3:** Quali-quantitative composition of CsA liposomes coated with CaP after dialysis. EE% = encapsulation efficiency; CsA loading = concentration of CsA obtained by HPLC.

	*Composition Post Dialysis*				
Batch (CsA Initial Conc.)	Solid Content (mg/mL)	CsA (mg/mL)	Ca^2+^ (mg/mL)	EE %	CsA Loading (% *w*/*w*)
A (4.8% *w*/*w*)	2.37 ± 0.08	0.14 ± 0.01	0.03 ± 0.01	100 ± 2.94	5.4 ± 0.01
B (7.0% *w*/*w*)	2.34 ± 0.12	0.20 ± 0.03	0.03 ± 0.02	95.2 ± 0.91	8.6 ± 0.7
C * (9.0% *w*/*w*)	1.57 ± 0.10	0.08 ± 0.05	N/A	27.6 ± 2.30	5.01 ± 0.3
D	1.99 ± 0.14	0.20 ± 0.02	0.02 ± 0.01	95.2 ± 2.31	9.9 ± 0.2
*E*	1.97 ± 0.04	0.18 ± 0.01	0.03 ± 0.01	85.7 ± 0.18	9.3 ± 0.2

* Precipitation of cyclosporine.

**Table 4:** Concentration of the components used for the production of the spray-dried powders, solid content concentration, yield of the process and the CsA content in the powder quantified by HPLC.

Powder	Liposome Concentration (mg/mL)	Mannitol (mg/mL)	Lubricant (mg/mL)	Solid Conc. (% *w*/*v*)	Yield (%)	CsA (% *w*/*w*)
LipoM-CsA_1	2.34 ± 0.12	2.34	-	0.5	3%	N/A
LipoM-CsA_2	2.34 ± 0.12	4.68	-	0.7	7%	NA
LipoM-CsA_3 *	2.34 ± 0.12	7.02	-	0.9	63%	1.59 ± 0.01
LipoM-CsA_4	2.34 ± 0.12	9.36	-	1.1	68%	1.19 ± 0.01
LipoM-Leu_CsA *	2.34 ± 0.12	5.97	1.05	0.9	65%	1.73 ± 0.08
LipoM-NaSt_CsA *	2.34 ± 0.12	6.98	0.03	0.9	70%	1.68 ± 0.01
ULipoM-Leu_CsA *	2.34 ± 0.12	5.97	1.05	0.9	65%	1.66 ± 0.01

* Powder batches further characterised.

**Table 5:** Aerodynamic characteristics of spray-dried powders produced with a 1:3 weight ratio between liposomes and excipients: coated liposomes in mannitol (LipoM-CsA_3), mannitol and leucine (LipoM-Leu-CsA), mannitol, leucine and sodium stearate (LipoM-NaSt-CsA), and mannitol and leucine embedding uncoated liposomes (ULipoM-Leu-CsA). MMAD = mass median aerodynamic diameter; FPM = fine particle mass; FPF = fine particle fraction.

Powder	Emitted Fraction (%)	MMAD (µm)	FPM (mg)	FPF (%)
LipoM_CsA_3	92.2 ± 0.3	9.88 ± 0.00	0.01 ± 0.01	3.3 ± 0.1
LipoM_NaSt-CsA	92.6 ± 4.2	10.09 ± 0.02	0.01 ± 0.01	**2.5 ± 2.5**
LipoM_Leu-CsA	77.8 ± 1.3	4.88 ± 0.03	0.08 ± 0.01	**33.6 ± 1.6**
ULipoM_Leu-CsA	75.9 ± 1.6	4.03 ± 0.16	0.12 ± 0.01	**50.5 ± 0.6**

**Table 6:** Size, PDI and Z-potential values relating to the reconstitution of LipoM_Leu-CsA powder spray-dried in buffer PBS, pH 7.4, and sodium acetate, pH 5.

	Size (d.nm)	PDI	ζ-Potential
Initial value	43.3 ± 5.7	0.19 ± 0.22	−27.9 ± 10.2
PBS (pH = 7.4)	176.0 ± 4.5	0.31 ± 0.34	−14.4 ± 0.5
Sodium acetate (pH = 5)	232.8 ± 2.5	0.34 ± 0.19	−12.9 ± 0.9

## Data Availability

The data presented in this study are available on request from the corresponding author.

## Abbreviations

The following abbreviations are used in this manuscript:

BOS	Bronchiolitis obliterans syndrome
CaP	Calcium phosphate
CsA	Cyclosporine A
Cryo-TEM	Cryogenic transmission electron microscopy
DPI	Dry powder for inhalation
ED	Emitted dose
FPD	Fine particle dose
FPF	Fine particle fraction
LPS	Lipopolysaccharide
MMAD	Mass median aerodynamic diameter
RF	Respirable fraction
SEM	Scanning electron microscopy
